# Electrical and chemical modulation of homogeneous and heterogeneous human-iPSCs-derived neuronal networks on high density arrays

**DOI:** 10.3389/fnmol.2024.1304507

**Published:** 2024-02-06

**Authors:** Giulia Parodi, Giorgia Zanini, Michela Chiappalone, Sergio Martinoia

**Affiliations:** Department of Informatics, Bioengineering, Robotics, and Systems Engineering (DIBRIS), University of Genova, Genoa, Italy

**Keywords:** human induced pluripotent stem cells, high density arrays, evoked response, electrophysiology, electrical stimulation, chemical stimulation, connectivity

## Abstract

The delicate “Excitatory/Inhibitory balance” between neurons holds significance in neurodegenerative and neurodevelopmental diseases. With the ultimate goal of creating a faithful *in vitro* model of the human brain, in this study, we investigated the critical factor of heterogeneity, focusing on the interplay between excitatory glutamatergic (E) and inhibitory GABAergic (I) neurons in neural networks. We used high-density Micro-Electrode Arrays (MEA) with 2304 recording electrodes to investigate two neuronal culture configurations: 100% glutamatergic (100E) and 75% glutamatergic / 25% GABAergic (75E25I) neurons. This allowed us to comprehensively characterize the spontaneous electrophysiological activity exhibited by mature cultures at 56 Days *in vitro*, a time point in which the GABA shift has already occurred. We explored the impact of heterogeneity also through electrical stimulation, revealing that the 100E configuration responded reliably, while the 75E25I required more parameter tuning for improved responses. Chemical stimulation with BIC showed an increase in terms of firing and bursting activity only in the 75E25I condition, while APV and CNQX induced significant alterations on both dynamics and functional connectivity. Our findings advance understanding of diverse neuron interactions and their role in network activity, offering insights for potential therapeutic interventions in neurological conditions. Overall, this work contributes to the development of a valuable human-based *in vitro* system for studying physiological and pathological conditions, emphasizing the pivotal role of neuron diversity in neural network dynamics.

## 1 Introduction

The human brain is characterized by several complex features that are pivotal for creating a faithful *in vitro* model of its functioning. These features encompass integration, modulation, three-dimensionality, and most importantly, heterogeneity (Brofiga et al., 2023). Heterogeneity, in this context, entails the existence of a variety of different neurons with distinct characteristics and functions. Within the human brain, particularly in regions like the cortex and hippocampus, the neuronal network primarily consists of two key types: glutamatergic neurons, constituting the 70–80% of the network, and GABAergic neurons, making up the remaining 20–30% (Sahara et al., 2012). These neurons play pivotal roles in orchestrating the neuronal communication, where glutamatergic neurons perform excitatory (E) functions, while GABAergic neurons undertake inhibitory (I) functions. The equilibrium, often referred to as the “E/I balance,” between these two neural counterparts is extremely fragile. Disruption of this balance can lead to the onset of debilitating neurodegenerative diseases, such as epilepsy or schizophrenia (Roberts, 1984; Grent-’t-jong et al., 2018) as well as neurodevelopmental and Autism Spectrum Disorders diseases (Pietropaolo and Provenzano, 2022). Despite the pivotal role of heterogeneity in these processes, it has received limited attention in models constructed using neuronal networks derived from human induced pluripotent stem cells (hiPSCs). Only recently, a study from our group demonstrated the feasibility and the stability of both homogeneous and heterogeneous neuronal cultures from hiPSCs with finely controlled E/I ratios (Parodi et al., 2023). In that study, we monitored and characterized the spontaneous electrophysiological activity of hiPSCs-derived neurons grown onto standard, low density Micro-Electrode Arrays (MEA) during their *in vitro* development.

By capitalizing on our (Parodi et al., 2023; Zanini et al., 2023) and others’ (Mossink et al., 2021a; Wang et al., 2023) previous works and by exploiting the recent advances in MEA technology, which now rely also on high density arrays (Amin et al., 2016; Ronchi et al., 2021), we here performed a step forward to provide the scientific community with a human-based *in vitro* system that holds significant potential for investigating a wide spectrum of physiological and pathological conditions.

Our study focused on two distinct neuronal culture configurations stably coupled to high-density MEAs with 2304 recording electrodes (3Brain GmbH). The first configuration consisted solely of pure putative glutamatergic neurons (referred to as 100E), while the second configuration involved a composition of 75% excitatory neurons and 25% inhibitory neurons (referred to as 75E25I). These configurations allowed us to explore the spontaneous electrophysiological activity in the mature networks (i.e., at 56 days *in vitro*), facilitating a comprehensive characterization of the neuronal cultures. We explored the impact of heterogeneity by performing electrical stimulation to see possible differential effects of the two configurations in terms of evoked activity. Finally, we examined how the introduction of drugs acting on glutamatergic and GABAergic neurons influenced both the dynamics and the functional connectivity of the monitored networks.

We found that the electrical stimulation was effective in producing reliable responses in the 100E configuration. However, in the case of the 75E25I, a more thorough phase of parameter tuning was required, since an increase of the current amplitude showed improvements in the evoked response, even if not comparable to the 100E configuration. As for chemical stimulation, BIC did not produce major changes to the dynamics and connectivity of the 100E networks, while APV and CNQX caused drastic alterations in both configurations.

Our results constitute an important step forward to unravel the intricate interplay between diverse neuron types and advance our understanding of how these interactions underlie network activity by means of high-density MEAs, thereby shedding light on potential therapeutic interventions for various neurological conditions.

## 2 Materials and methods

### 2.1 Ethical statements

The experimental protocol was approved by the European Animal Care Legislation (2010/63/EU), by the Italian Ministry of Health in accordance with the D.L. 116/1992 and by the guidelines of the University of Genova (Prot. 75F11.N.6JI, 08/08/18). We used two previously characterized hiPSCs lines (Mossink et al., 2021a). Both lines were generated from fibroblasts. Control line 1 (C1, healthy 30-years-old female) was reprogrammed via episomal reprogramming (Coriell Institute for medical research, GM25256). Control line 2 (C2, healthy 51-years-old male) was reprogrammed via a non-integrating Sendai virus (KULSTEM iPSC core facility Leuven, Belgium, KSF-16-025). We received the hiPSCs lines in frozen vials by Dr. Frega (University of Twente). We declare that the research was conducted in accordance with the principles embodied in the Declaration of Helsinki and in accordance with local statutory requirements. We declare that all participants gave written informed consent to participate in the study (Coriell Institute for medical research, GM25256 and KULSTEM iPSC core facility Leuven, Belgium, KSF-16-025). We declare that the research involves not identifiable human subjects, confirming that participant anonymity is totally protected.

### 2.2 HiPSCs cultures

**FIGURE 1:**
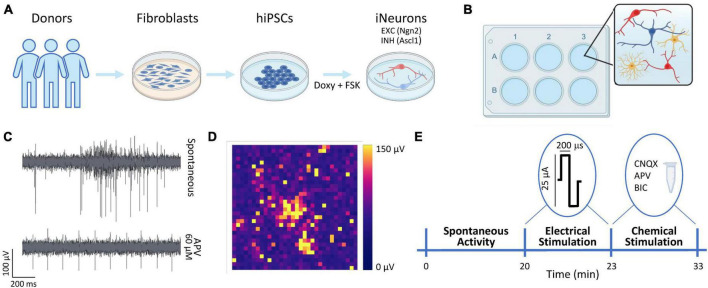
Overview of the experimental protocol. **(A)** Schematic representation of the neuronal cultures protocol. The hiPSCs were derived thanks to a reprogramming protocol applied to fibroblasts obtained from donors’ skin biopsies. The hiPSCs were induced to differentiate into excitatory (EXC, red) and inhibitory (INH, blue) neurons by adding doxycycline and forskolin (FSK) compounds to the culture medium. **(B)** The neurons (N, in red and blue) were moved to the 3Brain devices and mixed with rat astrocytes (As, in yellow) at the proportion equal to N:As 70:30. **(C)** Representative electrophysiological trace from a single electrode during spontaneous activity (top) and after the administration of APV (bottom). **(D)** Color map depicting the amplitude (in μV) of the electrophysiological activity of a representative neuronal network. Each pixel represents one channel. **(E)** Schematic representation of the experimental protocol. The spontaneous neuronal activity was recorder for 20 min. Subsequently, a 3-min stimulation train composed of 20 biphasic stimuli (at 0.1 Hz frequency) was emitted. Finally, a chemical stimulation was performed by adding either Bicuculine (BIC, 30 μM) or D-2-Amino-5-phosphonopentanoic acid (D-APV, 60 μM) or 6-cyano-7-nitroquinoxaline-2,3-dione (CNQX, 30 μM) to the medium and by recording for 10 min the neuronal activity after the drug administration.

We used two previously characterized hiPSCs lines genetically modified to obtain homogeneous populations of excitatory and inhibitory neurons (Figure 1A) thanks to the forced expression of the transcription factors Neurogenin-2 (Ngn2) and Achaete-scute homolog 1 (Ascl1) (Mossink et al., 2021a). We received the hiPSCs lines in frozen vials from Dr. Frega (University of Twente). Glutamatergic neurons were derived from control line 1 (C1, healthy 30-years-old female, Ngn2), while GABAergic neurons were derived from control line 2 (C2, healthy 51-years-old male, Ascl1). Cells were thawed and maintained in E8Flex medium (Thermo Fisher Scientific) with the following supplements: E8 supplements (2%, Thermo Fisher Scientific), penicillin/streptomycin (1%, Sigma-Aldrich), G418 (50 μg/ml, Sigma-Aldrich) and puromycine (0.5 μg/ml, Sigma-Aldrich). To differentiate the hiPSCs cultures into neuronal ones, the colonies were detached with ReLeSR (StemCell Technologies) and plated with the abovementioned supplemented medium enriched with RevitaCell (1%, Thermo Fisher Scientific) to favor the cells’ recovery, and with doxycycline (4 μg/ml, Sigma-Aldrich) and forskolin (4 μg/ml, Sigma-Aldrich) to induce the differentiation in early stage induced-neurons (iNs). The hiPSCs can be considered as differentiated into early stage neurons after about 3 days of doxycycline and forskolin treatment (Frega et al., 2017; Mossink et al., 2021a). The cultures were maintained in the incubator at stable condition (37°C, 5.5% CO_2_, 95% humidity atmosphere). The medium was refreshed at 50% every 2 days.

### 2.3 Neuronal network cultures

The neuronal networks were treated with the adapted protocol presented in Wang et al. (2023). Briefly, the 3Brain devices were sterilized in ethanol (70%, 1 h) and with UV light (45 min). The day before the cultures plating, the devices were pre-coated overnight with poly-L-Ornithine (50 μg/ml, Sigma-Aldrich) and human laminin (50 μg/ml, BioLamina) (Hyvärinen et al., 2019). At Day *in vitro* (DIV) 0, the neurons (iNs) were co-plated with rat astrocytes (As) to favor the neuronal growth and maturation (Banker, 1980; Tang et al., 2013; Bellot-Saez et al., 2018) in proportion equal to 70:30 (iNs:As) (Figure 1B). To obtain the homogeneous configuration (E:I 100:0), only glutamatergic neurons were plated, and we will refer to this configuration as 100E. To obtain the heterogeneous configuration, the glutamatergic and GABAergic neurons were mixed in proportion equal to E:I 75:25. We will refer to this configuration as 75E25I. The neuronal cultures density was equal to 1600 cells/mm^2^. During the first week, the neuronal cultures were maintained in Neurobasal medium (Thermo Fisher Scientific) with the following supplements: B27 supplements (2%, Thermo Fisher Scientific), penicillin/streptomycin (1%, Sigma-Aldrich), stable L-Glutamine (1% GlutaMax 100x, Thermo Fisher Scientific), human Brain-Derived Neurotrophic Factor (BDNF, 10 g/ml, Sigma-Aldrich), human Neurotrophin-3 (NT-3, 10 ng/ml, Sigma-Aldrich), doxycycline (4 μg/ml, Sigma-Aldrich) and forskolin (4 μg/ml, Sigma-Aldrich). After 7 DIV, Fetal Bovine Serum (FBS, 2%, Thermo Fisher Scientific) was added to the abovementioned supplemented medium to support astrocytes. After 14 DIV, doxycycline and forskolin were removed from the medium. The neuronal cultures on MEAs were maintained in the incubator in stable condition (37^°^C, 5.5% CO_2_, 95% humidity atmosphere) up to 59 DIV. The medium was refreshed at 50% each 2 days.

### 2.4 Set-up and electrophysiological recordings

The neuronal cultures were plated on commercial CorePlate 6-well devices (3Brain GmbH). The devices integrate 2304 electrodes for each well characterized by 60 μm in pitch and 25 μm in electrode size that allowed to record the extracellular signal traces (Figure 1C), thus providing high-resolution recordings of spiking activity all over the network. The 2304 electrodes are arranged in a 48 × 48 grid (Figure 1D). The electrophysiological recordings were performed at DIV 59 with the HyperCam System (3Brain GmbH). After a 10-min period of acclimatation out of the incubator, the spontaneous neuronal activity was recorder for 20 min in stable condition (37°C, 5% CO_2_) and sampled at 10 kHz. Subsequently, the neuronal cultures were stimulated with a biphasic (positive-then-negative) pulses characterized by a 25 or 35 μA amplitude (peak-to peak) and a duration of 200 μs. The stimuli were emitted with a frequency of 0.1 Hz, for a total of 20 stimuli for each stimulation session. The sites of stimulation for each well were chosen by selecting the electrodes that showed relevant spiking activity during the spontaneous-activity recordings. Finally, the chemical stimulation was performed by adding the chemical compounds to the medium, i.e., Bicuculine (BIC, 30 μM), to block GABA receptors, D-2-Amino-5-phosphonopentanoic acid (D-APV, 60 μM) to block NMDA receptors, and 6-cyano-7-nitroquinoxaline-2,3-dione (CNQX, 30 μM, Sigma-Aldrich) to block AMPA receptors. The electrophysiological activity was recorded for 10 min after the drugs administration. The protocol pipeline is summed up in Figure 1E.

### 2.5 Data analysis

In the following subsections, we present the analyses performed on the data and the features utilized to characterize the neuronal cultures. The features are summarized in the Glossary (Supplementary material).

#### 2.5.1 Spiking and bursting activity

Off-line data analysis was performed using BrainWave software (3Brain GmbH) and custom-made in-house codes developed in MATLAB (The Mathworks, Natick, MA, USA), to extract the parameters to describe the spontaneous network activity. In particular, spike detection was performed using the precision time spike detection (PTSD) algorithm (Maccione et al., 2009). The noise threshold for individual spike detection was set at 10 times the standard deviation of the baseline noise. The parameters required for the PTSD were the peak lifetime period (set at 2 ms), associated to the duration of the spike, and the refractory period (set at 2 ms), i.e., the minimum time elapsed between consecutive spikes. The mean firing rate (MFR, i.e., the number of spikes in the unit time) for each culture was calculated by averaging the firing rates of each active channel. A channel was considered active if its firing rate was greater than 0.1 spikes/second (Mossink et al., 2021b). Bursts were detected by setting a threshold of the neuronal inter-spike interval (ISI) and the minimum number of spikes belonging to a burst event. A burst was defined if at least 5 spikes occurred with an ISI lower than 100 ms. An active channel was considered as bursting if its bursting rate was greater than 0.4 bursts/minute (Mossink et al., 2021b). From the burst detection, we extrapolated the mean bursting rate (MBR, i.e., the number of bursts per minute), the burst duration (BD), and the percentage of random spikes (RS, i.e., the number of spikes not belonging to a burst). We defined an event as network burst if the following conditions were met: activity composed by at least 50 consecutive spikes within a 100 ms window, recruited from at least the 15% of the channels. From the network burst detection, the network burst duration (NBD) was computed.

#### 2.5.2 Cross-correlation-based analysis

Cross-correlation-based analysis were performed with BrainWave software (3Brain). Briefly, cross-correlation (CC) measures the frequency at which a neuron or electrode fires (target) as a function of time, in relation to the firing of an event in another one (reference). Given a reference electrode *x* and a target electrode *y*, the correlation function *Cxy(*τ*)* represents the probability of observing a spike in the train *y* at time (*t* + τ), given a spike in the train *x* at time *t*. The cross-correlogram is defined as the correlation function computed over a chosen correlation window (*W* = 100 ms) with a defined binning (bin size = 4 ms). A significant deviation in the cross-correlogram, i.e., a peak (excitatory link) or a trough (inhibitory link), is an indication of a functional excitatory or inhibitory connection. The different amplitude of the peaks can be related to the existence of different levels of synchronization between neural spike trains: we called this parameter *C*_*peak*_, and it is computed as the maximum value normalized between [0 and 1] of the cross-correlogram. We computed the *Time Delay* as the displacement of the evident one-sided peak of the cross-correlogram from the origin, that is an indication of the latency corresponding to the synaptic delay.

By considering the connections obtained from the cross-correlation-based analysis, the connectivity matrix was computed by performing a distribution-based thresholding, assuming as threshold μ + 2σ, where μ and σ represents the average and the standard deviation of the values, respectively. In this way, we obtained the connectivity graphs in which all the functional connections (edges) and the neurons involved (nodes) were reported. From the graphs, we computed several features to quantitatively explore the electrophysiological activity as the network topology varies. We computed the *Small World Index* (SWI) to evaluate the network level of integration and its topology by adopting the definition of Humphries and Gurney (2008). To quantify the number of the network-developed functional connections, we computed the average number of nodes and the number of links inside the networks. Finally, to characterize the degree of connectivity of each node, we considered the in-degree and the out-degree of each node, indicating the number of incoming and outcoming edges. We computed the total *Node Degree* as the sum of in- and out- degree for each node. Since the last three parameters (i.e., number of nodes, number of links and node degree) are related to the amount of the cells on the active area, we decided to normalize the values obtained from the chemical stimulation with respect to the spontaneous activity of the same network, thus computing the percentage of variation (Var) of such parameters.

#### 2.5.3 Evoked activity from electrical stimulation

To obtain quantitative information on the evoked neuronal activity we computed the Post-stimulus Time Histograms (PSTHs). The PSTHs were calculated by considering a 2.4 s time window after the stimulus emission. Specifically, we divided each time window into 4-ms bins and counted the number of spikes occurring in each time bin. Electrodes presenting PSTHs with an area (i.e., total number of spikes) lower than 4 were excluded from the analysis, as they were considered inactive. To determine the effectiveness of stimuli, we computed the percentage of stimuli capable of evoking a response out of a total of 20 stimuli. Moreover, to evaluate the responsiveness of the neuronal network, we calculated the area of the PSTH by considering a 1.2 s time window after the stimulus emission and the latency, representing the time between the emission of the stimulus and the first evoked spike.

#### 2.5.4 Statistical analysis

Statistical analyses were performed using MATLAB (The MathWorks, Natick, MA, USA). We evaluated the normal distribution of the data using the Kolmogorov-Smirnov normality test. Since the data were not normally distributed, we performed a non-parametric Kruskal-Wallis test. Concerning the chemical stimulation, our statistical tests assessed each condition in relation to the spontaneous activity, not involving comparisons between the two different configurations (i.e., 100E and 75E25I). Since each drug was tested on distinct MEAs, rendering each group independent from the others, we did not compare the effect of the different drugs among them. To establish statistically significant differences, *p*-values < 0.05 were considered significant. To minimize the risk of false positives, we adjusted the *p*-value exploiting the Bonferroni’s correction, by verifying every single hypothesis at a level of significance equal to α/η, where α is the level of statistical significance and η is the number of hypotheses. Asterisks over the plots show statistically significant differences with respect to the adjusted *p*-values. All values in the text are reported with mean ± standard deviation, unless otherwise stated.

## 3 Results

### 3.1 Spontaneous activity

**FIGURE 2:**
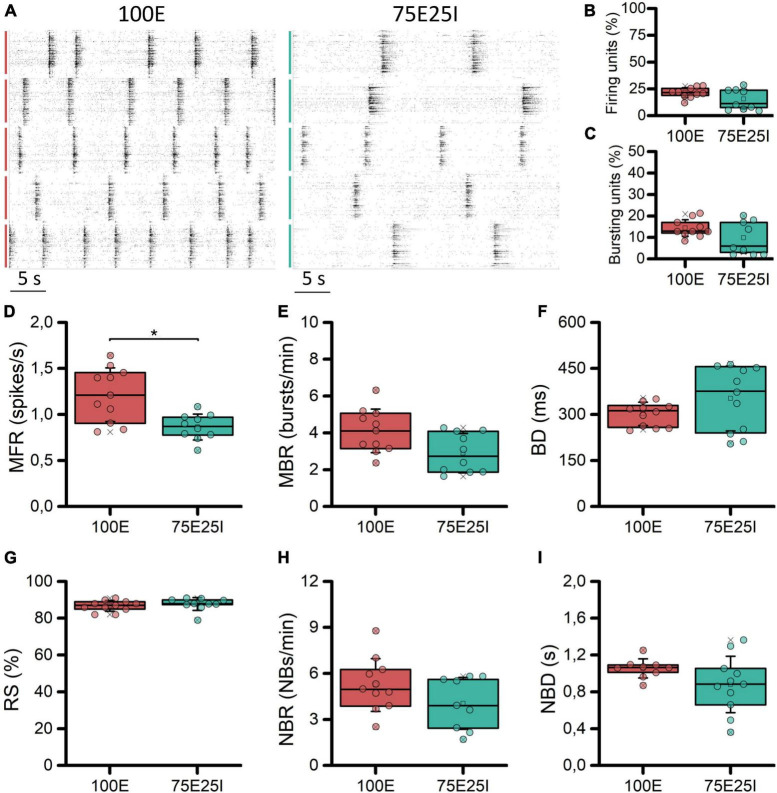
Characterization of the spontaneous electrophysiological activity. **(A)** Raster plots of 5-representative neuronal cultures of 100E (left) and 75E25I (right) configurations. Each spike is represented with a small black bar, while the bigger vertical lines indicate separate wells (red lines for the 100E, green lines for the 75E25I). Box plots of: **(B)** Active units: percentage of electrodes with a firing rate (MFR) greater than 0.1 spikes/s; **(C)** percentage of electrodes with a bursting rate (MBR) greater than 0.4 bursts/min; **(D)** Mean Firing Rate (MFR); **(E)** Mean Burst Rate (MBR); **(F)** Burst Duration (BD); **(G)** Percentage of random spikes (RS); **(H)** Network Burst Rate (NBR); and **(I)** Network Burst Duration (NBD) for the 100E (in red) and 75E25I (in green) configurations. In the box plots, data are represented with the 25th–75th percentile (box), the standard deviation (whiskers), the median (line), the mean (square), and the minimum and maximum (crosses) values. Raw data points are represented with circles over each box plot (* refers to *p* < 0.0083, adjusted with Bonferroni’s correction).

With the aim at characterizing the differential behavior of mature homogeneous (100E, *N* = 11) and heterogeneous (75E25I, *N* = 11) neuronal cultures stably coupled to high density MEAs, we firstly evaluated their spontaneous electrophysiological activity. By observing the raster plots of two representative multi-wells (Figure 2A), we qualitatively observed differences in the activity of the 100E and 75E25I. Specifically, the 100E exhibited a level of firing qualitatively higher than the 75E25I configuration. Moreover, the fully excitatory network was characterized also by sustained bursting activity. On the other hand, the duration of both bursts and network bursts event seemed to be comparable between the two configurations. To quantify this, we first computed the number of firing and bursting units to determine the degree to which the neuronal cultures were uniformly adhered and effectively coupled to the surface of the devices. The percentage of firing units (Figure 2B) was equal to the 21.61 ± 4.54% and 15.47 ± 9.05% for the 100E and 75E25I configurations, respectively, showing a comparable and satisfying adhesion of the cells on the devices, considering that the 20% of the high-density-device units correspond to more than 450 electrodes. The same result was obtained for the bursting units (Figure 2C) in which the 100E showed the 14.40 ± 3.86%, while the 75E25I showed 9.89 ± 7.22%. From a quantitative point of view, the differences previously observed in the raster plots were reflected by the computation of the MFR (Figure 2D and Supplementary Table 1) and the MBR (Figure 2E and Supplementary Table 1), in which the 100E showed higher values for both parameters with respect to the 75E25I configuration (Supplementary Tables 1, 2). Moreover, both the burst duration (BD, Figure 2F and Supplementary Table 1) and the percentage of the random spikes (RS, Figure 2G and Supplementary Table 1) did not show significant differences (Supplementary Table 2). While there was a noticeable difference in the network burst rate evident from the raster plot (Figure 2A) and the box plots (NBR, Figure 2H), no statistically significant differences emerged for this feature (Supplementary Table 2), as well as for the network burst duration (NBD, Figure 2I) between the two configurations.

### 3.2 Electrical stimulation

**FIGURE 3:**
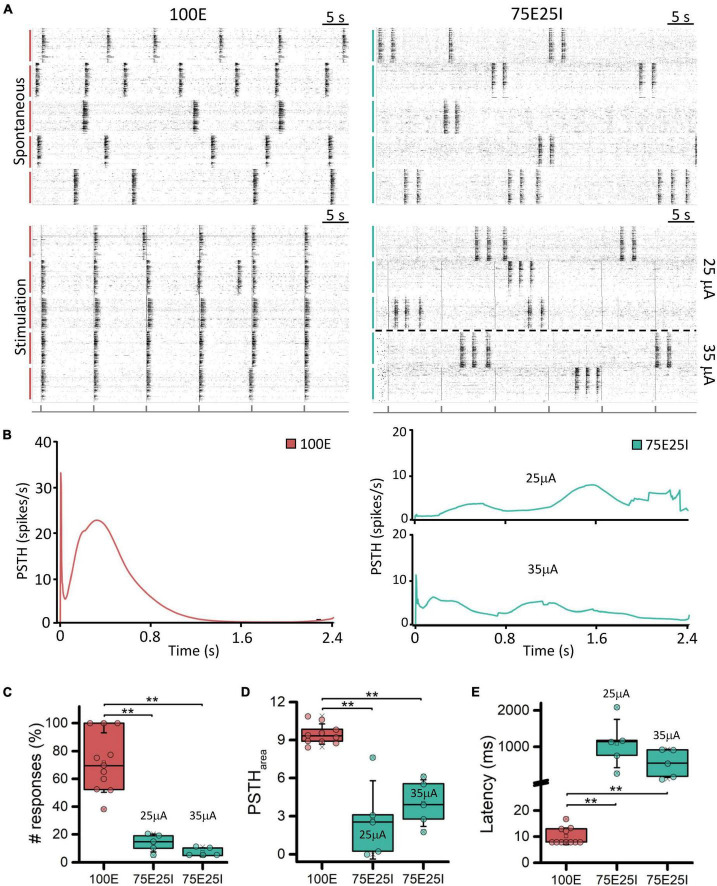
Qualitative and quantitative evoked response evaluation. **(A)** On the left: raster plots of 60-s of the spontaneous neuronal activity (on top) and evoked electrophysiological activity (on bottom) of the same 5-representative 100E neuronal networks, stimulated with a pulse amplitude of 25 mA. On the right: raster plots of 60-s of the spontaneous neuronal activity (on top) and evoked electrophysiological activity (on bottom) of the same 5-representative 75E25I neuronal networks, stimulated with a pulse amplitude of 25 mA (3 networks) and 35 mA (2 networks). Each spike is represented with a small black bar. On the bottom, a black line is reported to indicate the stimulus-emission timing. The vertical bars on the left of each raster plot represent different wells (red: 100E; green: 75E25I). **(B)** Average PSTH of the 100E (left) and 75E25I (right) neuronal networks. Box plots of: **(C)** Percentage of the positive responses to the stimulation; **(D)** PSTH area; **(E)** Latency of the first PSTH peak. In the box plots, data are represented with the percentile 25th–75th (box), the standard deviation (whiskers), the median (line), the mean (square), and the minimum and maximum (crosses) values. Raw data points are represented with circles over each box plot (** refers to *p* < 0.0034, adjusted with Bonferroni’s correction).

In order to better characterize our experimental models and to test their responsiveness to electrical stimulation, we applied current stimuli with different current amplitudes. Observing the raster plots in Figure 3A, the effect of electrical stimulation on the networks can be qualitatively appreciated. In particular, the 100E networks (*N* = 11) positively responded to all stimuli. Indeed, in the five recorded wells, the network activity was aligned at the emission of the stimulus, unlike the upper raster plot, where the cultures showed synchronous network events independently the one from the other during spontaneous activity. Regarding the 75E25I configuration (*N* = 10), the stimulation was not as effective as in the case of 100E, leaving the network activity almost unchanged without any evident synchronization with the emission of the stimuli even when using the highest current amplitude (i.e., 35 μA). Observing the PSTH (Figure 3B), the response of the 100E displayed a canonical shape, in which we appreciated an early response in the first ten of milliseconds characterized by a sharp peak. The early response was successively followed by a wide late response that ended around 1 s. Overall, the 100E PSTH outlined high recruitment and positive response from the network. On the other hand, the 75E25I showed an inconstant PSTH, characterized by an irregular shape of response, likely due to the baseline activity of the network itself, confirming what was qualitatively suggested the by raster plots, both at 25 μA (on bottom) and 35 μA (on top). The only appreciable difference was a hint of early and late response with the 35 μA stimulation, although smaller than the one evoked in 100E networks. Regarding the quantification of the number of responses (Figure 3C and Supplementary Table 3), the 100E responded on average at the 71.70% of the stimuli, therefore showing a good recruitment of the cultures. The latter observation was further confirmed by looking both at the 100E PSTH area (Figure 3D and Supplementary Table 3) and latency (Figure 3E and Supplementary Table 3). On the other hand, the 75E25I responded to the 13.83 and 7.26% of the stimuli when stimulated with 25 and 35 μA, respectively, and no significant differences were observed between the used amplitude (Supplementary Table 4). However, 35 μA stimulation elicited a slightly higher (Figure 3D and Supplementary Table 3) and shorter (Figure 3E and Supplementary Table 3) response than the 25 μA.

### 3.3 Chemical stimulation

**FIGURE 4:**
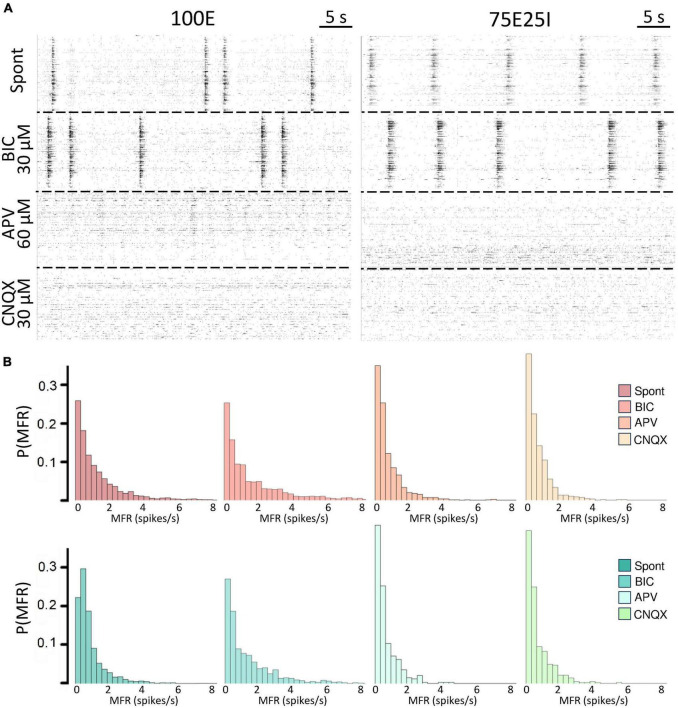
Qualitative evaluation of the neuronal activity after the drugs administration. **(A)** Raster plots of 60-s of the electrophysiological activity of the same representative 100E (left) and 75E25I (right) neuronal network, after the drug administration of BIC, APV and CNQX. Each spike is represented with a small black bar. **(B)** Distribution of the MFR of 100E (top) and 75E25I (bottom) neuronal cultures, during spontaneous activity and after the administrations of the drugs.

**FIGURE 5:**
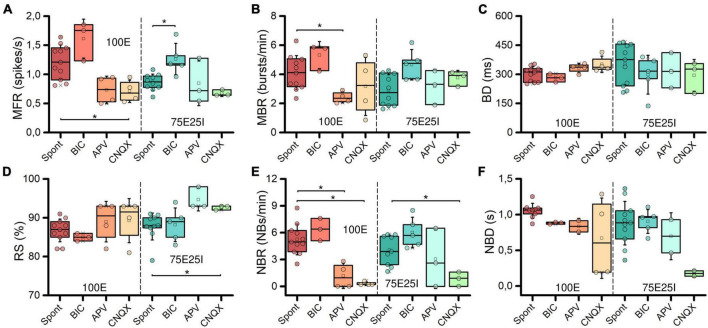
Characterization of the neuronal activity after the drugs administration. Box plots of: **(A)** Mean Firing Rate (MFR); **(B)** Mean Bursting Rate (MBR); **(C)** Burst Duration (BD); **(D)** Percentage of random spikes (RS); **(E)** Network Burst Rate (NBR); **(F)** Network Burst Duration (NBD). In the box plots, data are represented with the percentile 25th–75th (box), the standard deviation (whiskers), the median (line), the mean (square), and the minimum and maximum (crosses) values. Raw data points are represented with circles over each box plot (* refers to *p* < 0.0167, adjusted with Bonferroni’s correction).

To further explore the dynamics of our neuronal networks’ configurations (i.e., the 100E and the mixed 75E25I), we analyzed the responses triggered by chemical stimulation. By using CNQX (*N*_100*E*_ = 4, *N*_*75E25I*_ = 3) and APV (*N*_100*E*_ = 4, *N*_*75E25I*_ = 3), which are blockers of AMPA and NMDA receptors, respectively, we observed a significant reduction in firing and network bursting activity in both configurations (Figure 4A). However, upon an initial qualitative assessment, it appeared that BIC did not produce noticeable changes with respect to the spontaneous activity (*N*_*100E*_ = 3, *N*_*75E25I*_ = 5, Figure 4A). To precisely determine the extent to which these drugs influenced neuronal activity, we computed the distribution of firing patterns for both configurations (Figure 4B). After the administration of BIC, the 100E neuronal networks exhibited a similar pattern to the distribution of spontaneous firing. The 75E25I did not show great differences, except for a small shift in the peak (from the second to the first bin) and a slight increase in the bins associated with higher firing rates. A noticeable shift toward lower firing rates is evident for both configurations upon administration of CNQX and APV. This shift is characterized by a distinct peak in the range of MFR values between 0 and 0.4 spikes/s. In quantitative terms, the MFR (Figure 5A and Supplementary Tables 5, 7) showed lower values with respect to the spontaneous activity upon administration of CNQX for both configurations (Supplementary Tables 6, 8). Similarly, the APV caused a decreasing in the firing activity, despite not showing statistical differences with respect to the spontaneous condition (Supplementary Tables 6, 8). On the other hand, BIC significantly increased the firing rate in the 75E25I configuration (*p* = 0.0071). An analogous trend can be observed for the MBR (Figure 5B and Supplementary Tables 5, 7) of both configurations, while burst duration was not affected by the administration of the drugs (Figure 5C and Supplementary Tables 5, 7). Interestingly, the RS (Figure 5D and Supplementary Tables 5, 7) showed an increasing trend for both configurations when CNQX and APV were administered, indicating a dispersion of burst events within the neuronal networks. Indeed, the major significant differences arose with the network burst rate (NBR, Figure 5E and Supplementary Tables 5–8): CNQX and APV completely abolished the network burst events, leading to a NBR close to zero, especially upon CNQX administration. As a consequence, also the NBD was reduced (Figure 5F and Supplementary Tables 5, 7).

### 3.4 Functional connectivity

**FIGURE 6:**
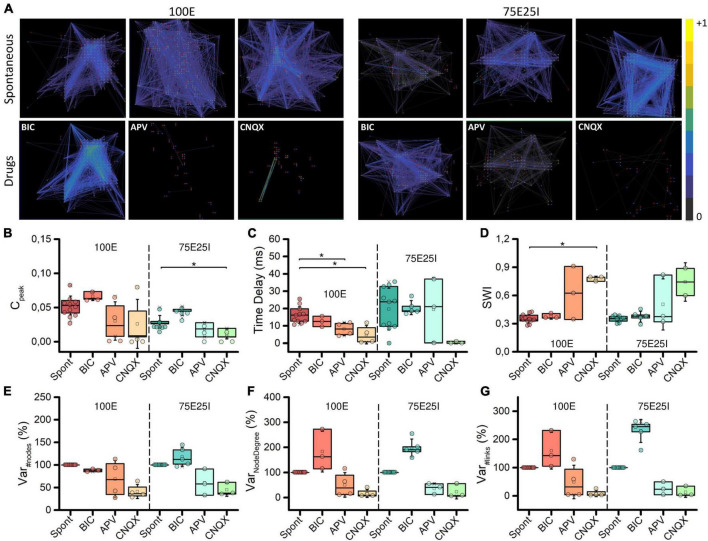
Topology and connectivity characterization. **(A)** Connectivity graphs of representative 100E (left) and 75E25I (right) neuronal cultures, during the spontaneous activity and after the administrations of drugs, i.e., CNQX, APV and BIC (from left to right). Each node is represented by dots (red: sender node, blue: receiver node, gray: broker node) and functional connections are represented with edges. Box plots of: **(B)** Maximum of the cross-correlogram (C_peak_); **(C)** Time Delay; **(D)** Swall World Index (SWI); **(E)** Number of Nodes; **(F)** Node degree; **(G)** Number of Links. In the box plots, data are represented with the percentile 25th–75th (box), the standard deviation (whiskers), the median (line), the mean (square), and the minimum and maximum (crosses) values. Raw data points are represented with circles over each box plot (* refers to *p* < 0.0167, adjusted with Bonferroni’s correction).

To deepen the synaptic physiology of the hiPSCs-derived neuronal networks, we investigated the topology of the graphs and the effects of the chemical compounds administration on the functional connectivity (Figure 6A). For what concerns the graphs obtained from the spontaneous activity analysis, the topology displayed a qualitative scale-free organization: a few highly connected nodes (hubs) to many others were present, self-organizing and producing branched structures for both 100E and 75E25I configurations. With respect to the spontaneous condition, BIC administration did not cause significant changes in the topology of the graph, but tended to increase the strength of the connections, as can be seen from the more intense color of the graph edges of both configurations (Figure 6A). The CNQX induced a clear variation of the connectivity for both the configurations, drastically modifying the topology and reducing both the number of links and nodes. Similar considerations may be asserted for the use of APV, even if it brought to a lower decrease in nodes and links compared to CNQX. To quantify the above obtained qualitative results, we computed the cross-correlation index C_*peak*_ (see section “Materials and methods”). We found that C_*peak*_ values during spontaneous activity were almost comparable to those obtained during BIC administration for both the 100E and the 75E25I (i.e., no statistical difference between Spont and BIC, Figure 6B and Supplementary Tables 9–12). On the contrary, C_*peak*_ average values during spontaneous activity were higher than those calculated for CNQX and APV (Figure 6B and Supplementary Tables 9, 11). The Time Delay (Figure 6C), indicator of the speed of information transmission, showed lower values with the use of drugs, but only statistically significant with the use of CNQX and APV in the case of 100E (Supplementary Tables 10, 12). After causing a significant shift in topology, the reduction in the C_*peak*_ value (indicative of enhanced connectivity) resulted in a higher Small World Index (SWI, Figure 6D) in both configurations after the use of CNQX and APV, albeit consistently significant only with CNQX use for 100E configuration (Supplementary Tables 10, 12). This suggested that the remaining connections following drug administration exhibited a topology more closely resembling small-world organization. In parallel to the above, the percentage of variation of the number of nodes was drastically reduced after the use of CNQX and APV (Figure 6E). These drugs had a disruptive impact on network connectivity, leading to a reduction not only in the number of nodes (Figure 6E), but also in the number of links (Figure 6G). The remaining nodes were unable to compensate for the decrease in node count resulting in a simultaneous decline in the Node Degree (Figure 6F). Conversely, the use of BIC resulted in a comparable number of nodes. Nevertheless, the network was able to establish strong connectivity, leading to an increase in Node Degree (Figure 6F) and in the number of links (Figure 6G) compared to the spontaneous phase.

## 4 Discussion

In this study, we investigated both activity and functional connectivity of human induced pluripotent stem cells-derived neuronal networks, specifically homogeneous networks (comprising 100% excitatory neurons, denoted as 100E) and heterogeneous networks (composed of 75% excitatory and 25% inhibitory neurons, denoted as 75E25I). Our primary objective was to gain a comprehensive understanding of these networks, with a specific focus on elucidating the significance of heterogeneity. To achieve that goal, we utilized high-density micro-electrode arrays to widely analyze the electrophysiological activity of both the homogeneous and heterogeneous neuronal networks. We carried out our experiments at DIV 56 as the shift of GABA occurs after about DIV42 (Mossink et al., 2021a). Our investigation encompassed the assessment of spontaneous and evoked neuronal activity, induced by both electrical and chemical modulation.

We demonstrated that the cells adhered effectively and in reasonable numbers to the surface of high-density devices, showing more than 350 active electrodes. Regarding spontaneous electrophysiological activity, we observed that homogeneous networks exhibited a higher firing and bursting activity compared to heterogeneous networks. This contrasts with findings from low-density devices, such as the 60-electrodes MEA, in a previous study (Parodi et al., 2023). Our result might be due to the nature of high-density devices, which are smaller in size and capable of recording activity at the single-cell level. This capability allowed us to derive parameters that differ from those observed using traditional MEAs.

We then evaluated the responsiveness of neuronal cultures to electrical stimulation on both configurations, i.e., 100E and 75E25I. Our findings revealed notable differences between these configurations. The 100E networks exhibited a strong and synchronized response to electrical stimuli, with a characteristic PSTH shape, indicating high network recruitment. Additionally, these human-derived networks displayed much longer responses, running out in about 1.2 s, which is in contrast with the responses typically observed in primary rodent-derived networks, lasting 500–600 ms (Chiappalone et al., 2008; Vajda et al., 2008; Le Feber et al., 2010; Scarsi et al., 2017). On the other hand, the 75E25I networks showed low responsiveness, even at the highest current amplitude. Further investigations are needed to elucidate the underlying mechanisms governing these differences and to optimize stimulation protocols for specific network configurations.

Regarding chemical stimulation, we assessed the *in vitro* neuronal activity following the administration of drugs. We investigated the effects on both excitatory neurotransmission (achieved by reducing glutamatergic signaling through the application of APV and CNQX) and inhibitory neurotransmission (achieved by reducing GABAergic signaling through BIC application). In line with the results obtained with rodent-derived and human-embryonic-stem-cells-derived neurons (Bonzano et al., 2006; Ylä-Outinen et al., 2010), we showed that APV and CNQX mainly reduced the global activity of the network, by suppressing the network bursting activity, resulting in a few isolated spikes. On the other hand, as expected, the application of BIC did not show significant differences with respect to the spontaneous activity for 100E configuration, while it caused an increase in the firing and bursting activity of the 75E25I networks.

Previous studies have conducted toxicity tests and characterizations using human iPSCs coupled to low-density devices (Odawara et al., 2016; Frega et al., 2019; Hyvärinen et al., 2019; Kang et al., 2022), which have limited the depth of analysis. Indeed, these devices lack the capacity for detailed investigations, such as connectivity analysis, which is essential for understanding network synaptic dynamics. In contrast, the utilization of high-density devices, as demonstrated in a rat model (Ullo et al., 2014), offers a significant advantage. In our study, we used high-density micro-electrode arrays which provide cellular resolution, enabling a comprehensive exploration of functional connectivity at the level of individual cells. We were then able to build the functional maps of our networks and extract several graph-related metrics to highlight their functional and topological organization. We found that BIC influenced the connectivity of 75E25I networks, by increasing the number of links and the Node Degree, while APV and CNQX did affect the connectivity of both configurations, by globally reducing the strength and the number of links. Notwithstanding, the Small World Index reached the maximum values for CNQX, indicating that, in presence of more random spiking, the small world organization is more likely to happen. This can suggest that a lower level of bursting activity can be the key to create more plausible *in vitro* system, resembling the functional connectivity and the properties observed in the human brain (Watts and Strogatz, 1998; Bassett et al., 2006; Caliandro et al., 2017).

In conclusion, hiPSCs-derived neuronal networks coupled to high density array can pave the way for a more comprehensive exploration of complex neural circuits. This enhanced understanding of low-level functions will be instrumental for elucidating higher-level brain behaviors, both in physiological and pathological conditions. Specifically, with a focus on neurodevelopmental diseases associated with an E/I imbalance (Pietropaolo and Provenzano, 2022), our networks can be utilized to simulate the initial phases of development, serving as a robust model for the investigation of these disorders.

## Data availability statement

The raw data supporting the conclusions of this article will be made available by the authors, without undue reservation.

## Ethics statement

Ethical approval was not required for the studies on humans in accordance with the local legislation and institutional requirements because only commercially available established cell lines were used. The animal study was approved by the experimental protocol was approved by the European Animal Care Legislation (2010/63/EU), by the Italian Ministry of Health in accordance with the D.L. 116/1992 and by the guidelines of the University of Genova (Prot. 75F11.N.6JI, 08/08/18). The study was conducted in accordance with the local legislation and institutional requirements.

## Author contributions

GP: Conceptualization, Data curation, Formal Analysis, Investigation, Methodology, Visualization, Writing – original draft, Writing – review and editing. GZ: Formal Analysis, Visualization, Writing – review and editing. MC: Conceptualization, Supervision, Validation, Writing – review and editing. SM: Conceptualization, Resources, Supervision, Validation, Writing – review and editing.
