# Supplementary material for: Electrical and chemical modulation of homogeneous and heterogeneous human-iPSCs-derived neuronal networks on high density arrays
Source: Front Mol Neurosci. 2024 Feb 6;17:1304507. doi: 10.3389/fnmol.2024.1304507 (PMC10877635; doi:10.3389/fnmol.2024.1304507)
Supplement: Supplementary file 1 [file Data_Sheet_1.PDF]

Electrical and chemical modulation of homogeneous and heterogeneous human-iPSCs-derived neuronal networks on high density arrays

Giulia Parodi, Giorgia Zanini, Michela Chiappalone, and Sergio Martinoia

Supplementary Material

Glossary

Glossary of the main parameters used to quantify the electrophysiological activity.

Parameter	***Short Name***	***Unit***	***Explanation***
Burst Duration	BD	**s**	The BD represents the duration of the bursts.
Inter Spike Interval	ISI	s	The ISI represents the temporal distance between two consecutive spikes.
Mean Bursting Rate	MBR	bursts/min	The MBR represents the number of bursts per minute.
Mean Firing Rate	MFR	spikes/s	The MFR represents the number of spikes in the unit time.
Mean Network Bursting Rate	NBR	network bursts/min	The NBR represents the number of network bursts detected in a minute.
Network Burst Duration	NBD	s	The NBD represents the duration of the network bursts.
Post Stimulus Time Histogram	PSTH	spikes/s	The PSTH represents the rate of spikes in relation to an external stimulus.
Random Spikes	RS	%	The RS represents the percentage of spikes that do not belong to a burst.
Small World Index	SWI	**-**	The SWI represents the level of the integration of the network.

	100E	75E25I
Firing units	21.61 ± 4.54	15.47 ± 9.05
Bursting units	14.4 ± 3.86	9.89 ± 7.22
MFR	1.21 ± 0.29	0.86 ± 0.14
MBR	4.11 ± 1.18	2.90 ± 1.07
BD	301.82 ± 37.83	352.34 ± 105.44
RS	86.73 ± 2.94	87.75 ± 3.47
NBR	5.25 ± 1.72	4.05 ± 1.69
NBD	1054.02 ± 104.41	879.87 ± 305.34

Table S1: Features' average values (mean values ± standard deviation) for both configurations of firing units, bursting units, MFR, MBR, BD, RS, NBR, and NBD.

	Adjusted p-value
MFR	**0.0075**
MBR	0.0346
BD	0.4813
RS	0.3582
NBR	0.3979
NBD	0.0783

Table S2: P-values adjusted with Bonferroni correction with $\eta = 6$ (significative differences with $p < 0.0083$ are marked in bold) for MFR, MBR, BD, RS, NBR, and NBD comparing 100E vs 75E25I configuration.

	100E	75E25I	
	25 μA	25 μA	35 μA
# responses	71.70 ± 21.32	13.83 ± 6.38	7.26 ± 3.11
$PSTH_{area}$	9.48 ± 0.80	2.71 ± 3.08	4.04 ± 1.83
Latency	10.14 ± 3.16	1091 ± 662.22	545.17 ± 384.64

Table S3: Average values (mean values ± standard deviation) for both configurations (100E and 75E25I) for: number of responses (# responses), PSTH area ($PSTH_{area}$), and Latency.

	$100E_{25\mu A}$ vs $75E25I_{25\ \mu A}$	$100E_{25\mu A}$ vs $75E25I_{35\ \mu A}$	$75E25I_{25\mu A}$ vs $75E25I_{35\ \mu A}$
# responses	**0.0018**	**0.0017**	0.1314
$PSTH_{area}$	**0.0022**	**0.0022**	0.3472
Latency	**0.0011**	**0.0011**	0.1172

Table S4: P-values adjusted with Bonferroni correction with $\eta = 3$ (significative differences with $p < 0.0167$ are marked in bold) for the number of responses, the area of PSTH and the latency.

	100E			
	Spont	BIC	APV	CNQX
MFR	1.21 ± 0.29	1.61 ± 0.34	0.73 ± 0.24	0.71 ± 0.19
MBR	4.11 ± 1.18	5.32 ± 0.93	2.42 ± 0.39	3.17 ± 2.00
BD	301.82 ± 37.83	281.67 ± 21.50	332.25 ± 24.03	351.75 ± 42.38
RS	86.73 ± 2.94	85 ± 1	89 ± 5.23	89.25 ± 5.68
NBR	5.25 ± 1.72	6.37 ± 1.25	1.18 ± 1.41	0.30 ± 0.2
NBD	1054.02 ± 104.41	881.12 ± 14.44	835.63 ± 111.31	670.05 ± 562.51

Table S5*:* Average values (mean values ± standard deviation) of dynamics-related features for 100E configuration during the spontaneous phase and during each drug administration phase.

	100E		
	Spont vs BIC	Spont vs APV	Spont vs CNQX
MFR	0.0734	0.0367	**0.0131**
MBR	0.1391	**0.0090**	0.4334
BD	0.4835	0.1510	0.0777
RS	0.2094	0.2919	0.2128
NBR	0.1857	**0.0061**	**0.0040**
NBD	0.0734	0.0757	0.2400

Table S6: P-values adjusted with Bonferroni correction with $\eta = 3$ (significative differences with $p < 0.0167$ are marked in bold) for each drug administration phase for the dynamics-related features of 100E configuration.

	75E25I			
	Spont	BIC	APV	CNQX
MFR	0.86 ± 0.14	1.26 ± 0.27	0.85 ± 0.38	0.67 ± 0.05
MBR	2.90 ± 1.07	4.66 ± 1.05	3.16 ± 1.18	3.76 ± 0.52
BD	352.34 ± 105.44	298 ± 100.42	319 ± 91.07	294.33 ± 82.03
RS	87.75 ± 3.47	88.20 ± 4.32	94.67 ± 2.89	92.67 ± 0.58
NBR	4.05 ± 1.69	6.02 ± 1.71	3.03 ± 3.27	0.83 ± 0.80
NBD	879.87 ± 305.34	904.44 ± 167.85	696.55 ± 328.74	175.68 ± 53.59

Table S7: Average values (mean values ± standard deviation) of dynamics-related features for 75E25I configuration during the spontaneous phase and during each drug administration phase.

	75E25I		
	Spont vs BIC	Spont vs APV	Spont vs CNQX
MFR	**0.0071**	0.0532	0.0425
MBR	0.0275	0.8299	0.2367
BD	0.4624	0.3902	0.3105
RS	0.9511	0.0341	**0.0111**
NBR	0.2203	0.1319	**0.0111**
NBD	0.6242	0.2059	0.0317

Table S8: P-values adjusted with Bonferroni correction with $\eta = 3$ (significative differences with $p < 0.0167$ are marked in bold) for the dynamics-related features of 75E25I configuration.

	100E			
	Spont	BIC	APV	CNQX
C_{peak}	0.05 ± 0.01	0.07 ± 0.01	0.03 ± 0.03	0.03 ± 0.04
Time Delay	16.30 ± 4,81	12.55 ± 3,03	8.15 ± 3,79	4.85 ± 5,28
SWI	0.35 ± 0.05	0.38 ± 0.03	0.63 ± 0.28	0.78 ± 0.03
$Var_{\#nodes}$	100 ± 0	87.99 ± 2.98	68.84 ± 40.78	41.25 ± 15.62
$Var_{NodeDegree}$	100 ± 0	183.13 ± 80.89	50.65 ± 49.35	16.29 ± 16.03
$Var_{\#links}$	100 ± 0	159.87 ± 65.20	49.15 ± 59.54	8.50 ± 11.39

Table S9: Average values (mean values ± standard deviation) of connectivity-related features for 100E configuration during the spontaneous phase and during each drug administration phase.

	100E		
	Spont vs BIC	Spont vs APV	Spont vs CNQX
C_{peak}	0.0734	0.1510	0.1172
Time Delay	0.1391	**0.0131**	**0.0090**
SWI	0.1857	0.0676	**0.0102**

Table S10: P-values adjusted with Bonferroni correction with $\eta = 3$ (significative differences with $p < 0.0167$ are marked in bold) for the connectivity-related features of 100E configuration.

	75E25I			
	Spont	BIC	APV	CNQX
C_{peak}	0.03 ± 0.01	0.04 ± 0.01	0.02 ± 0.01	0.01 ± 0.01
Time Delay	19.68 ± 12,08	20.54 ± 4,08	19.43 ± 18,49	0.35 ± 0,61
SWI	0.35 ± 0.03	0.37 ± 0.06	0.50 ± 0.27	0.74 ± 0.21
$Var_{\#nodes}$	100 ± 0	117.66 ± 20.56	60.75 ± 29.16	45.03 ± 14.57
$Var_{NodeDegree}$	100 ± 0	197.59 ± 35.54	35.91 ± 21.80	22.98 ± 28.32
$Var_{\#links}$	100 ± 0	230.24 ± 40.74	25.79 ± 23.04	13.09 ± 18.47

Table S11: Average values (mean values ± standard deviation) of connectivity-related features for 75E25I configuration during the spontaneous phase and during each drug administration phase.

	75E25I		
	Spont vs BIC	Spont vs APV	Spont vs CNQX
C_{peak}	0.4624	0.2207	**0.0112**
Time Delay	0.9468	0.2386	0.1172
SWI	0.5485	0.6374	0.0339

Table S12: P-values adjusted with Bonferroni correction with $\eta = 3$ (significative differences with $p < 0.0167$ are marked in bold) for the connectivity-related features of 75E25I configuration.
